# Typical MRI Features of a Vein of Galen Aneurysmal Malformation

**DOI:** 10.5334/jbsr.2133

**Published:** 2020-06-22

**Authors:** Damienne Vande Berg, Richard Pitcher, Dana Dumitriu

**Affiliations:** 1 UCL, BE; 2 Stellenbosch University, ZA

**Keywords:** aneurysmal malformation, congenital, vein of Galen, heart failure, pediatric

## Abstract

**Teaching Point:** Typical imaging features of a vein of Galen aneurysmal malformation are enlarged intracranial arterial feeders to a dilated recipient vein.

## Case History

**Figure 1:**
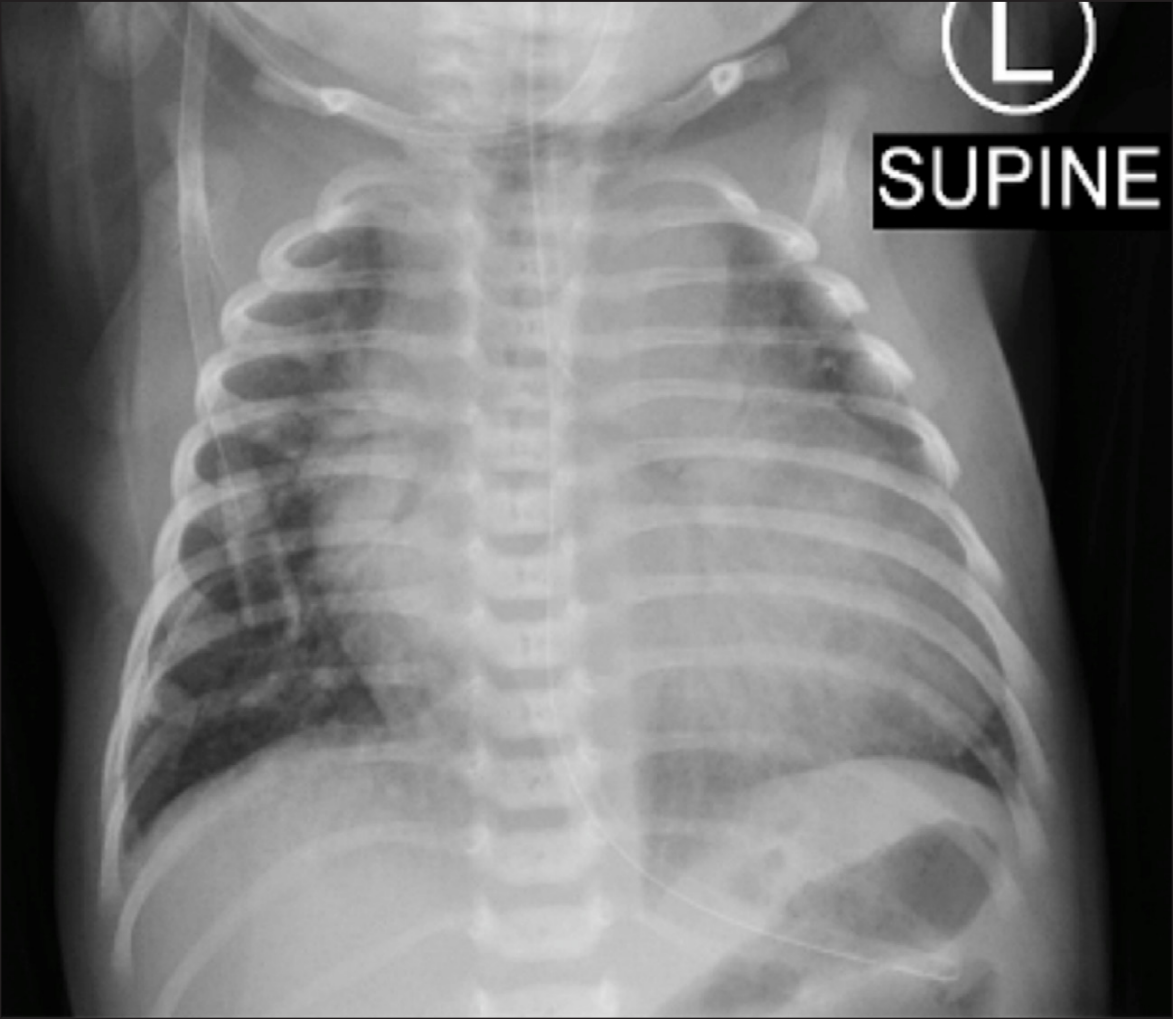


**Figure 2:**
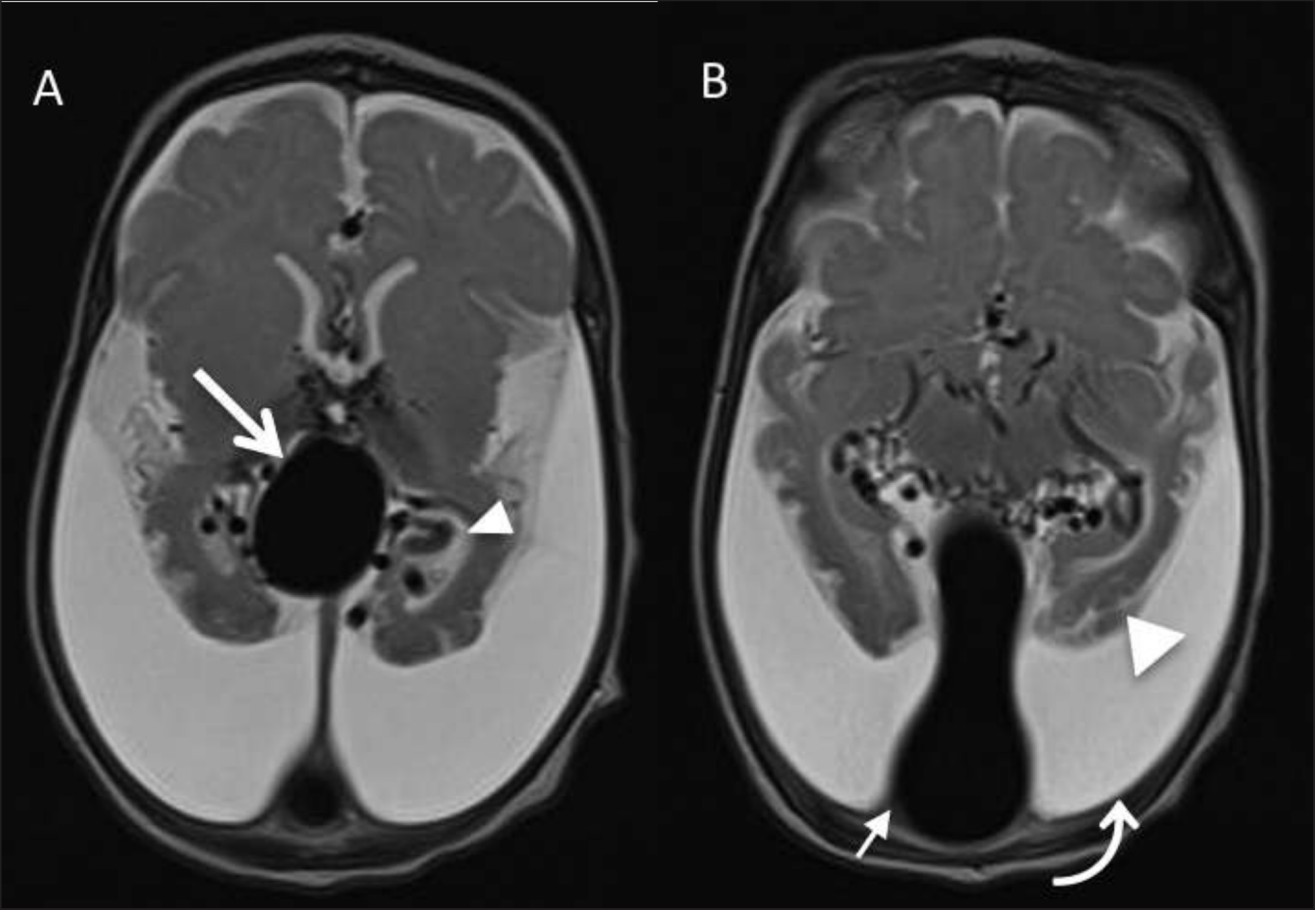


**Figure 3:**
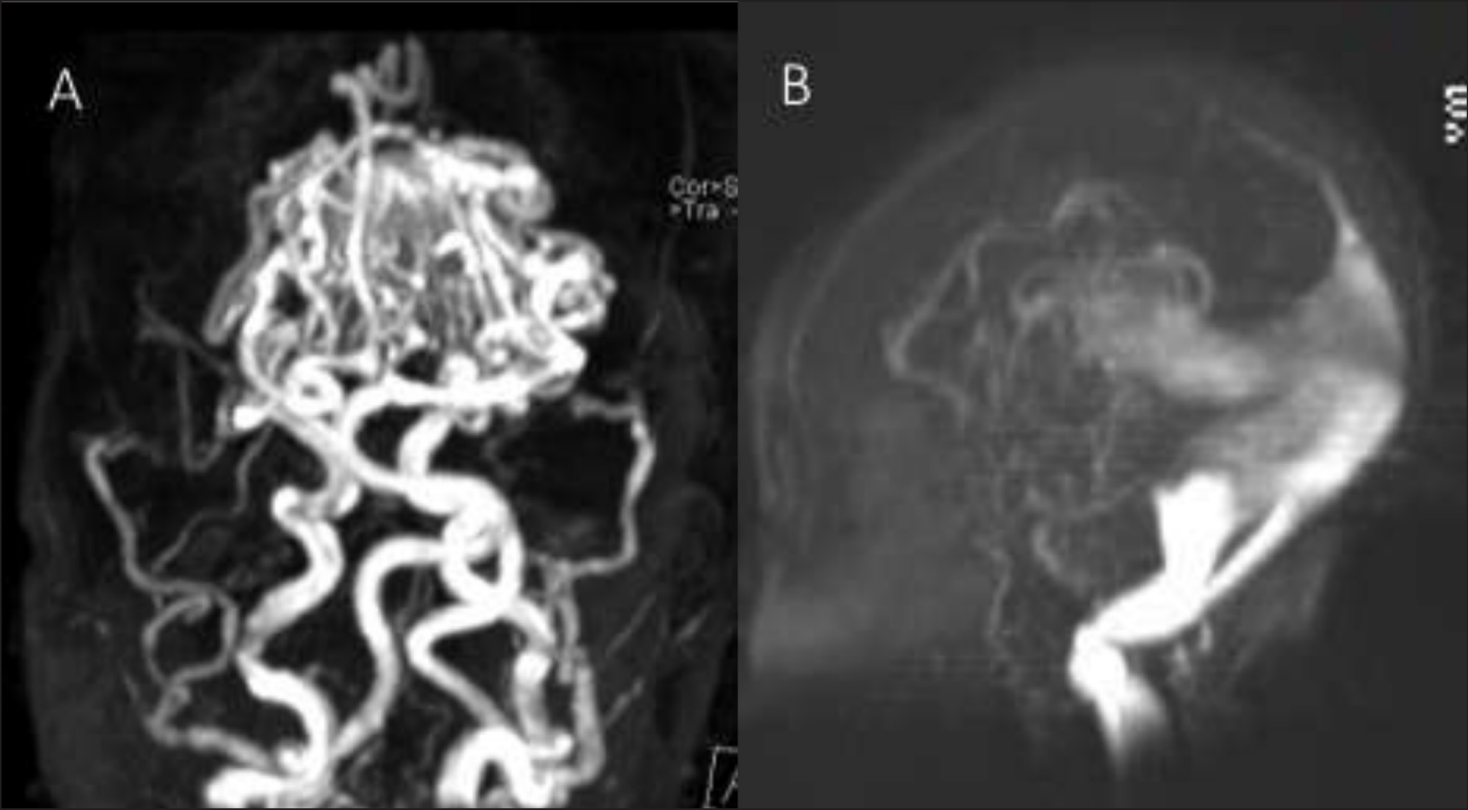


A newborn with congestive cardiac failure, increased head circumference and cranial bruits was transferred from a primary care center for the evaluation of a prenatally diagnosed cerebral vascular malformation. Chest radiograph (Figure 1) revealed cardiomegaly and pulmonary congestion. Brain magnetic resonance imaging (MRI) (Figure 2a and 2b – axial T2-weighted images) showed a well-circumscribed, markedly T2 hypointense, oval, midline mass (41 × 35 × 34 mm), dorsal to the third ventricle (large arrow), in continuity with a dilated inferior sagittal sinus (small arrow). Additionally, numerous serpiginous T2 hypointense foci (small arrowhead), consistent with flow voids, surrounded the midline lesion. The particularity in this case was the lack of hydrocephalus; instead, there was a marked bilateral cerebral atrophy, involving the parietal, occipital, and temporal lobes (large arrowhead). Large bilateral chronic subdural collections (curved arrow) were associated, representing the fluid-filled empty space formed by the cerebral atrophy and the often-large pericerebral space in infants. Time-of-flight MR angiography (Figure 3a and 3b–3D TOF images) revealed the early arterial filling of the venous complex and depicted the complex proliferation of abnormal arterial channels arising from the Circle of Willis, corresponding to the prominent flow voids on the T2-WI. No abnormality was noted on diffusion-weighted images. The MRI features were actually consistent with a vein of Galen aneurysmal malformation (VGAM). In this case, no further diagnostic or therapeutic steps were undertaken, given the bad prognosis even in case of successful embolization.

## Comment

VGAMs are rare embryogenic vascular malformations occurring between the 6th–11th week of gestation. They are characterized by arteriovenous fistulas between primitive choroidal arteries and the median prosencephalic vein, the embryonic precursor to the vein of Galen, with subsequent enlargement of the arteriovenous system. The fistulas prevent regression of the precursor to the vein of Galen and prohibit the development of the latter. The malformation causes a left-to-right shunt resulting in high cardiac output failure.

In the literature, different types are described based on arterial feeders, location of the fistulas, and degree of venous ectasia [1]. The most common choroidal type is characterized by numerous bilateral and symmetric connections mainly from the anterior choroidal arteries and accessorily from the pericallosal and thalamoperforating vessels to the anterior wall of the prosencephalic vein. The mural type is defined by fewer but larger unilateral or bilateral connections involving most commonly the posterior choroidal or collicular arteries.

The malformation mostly presents in neonates and infants with signs of congestive heart failure, cranial bruits, and craniomegaly. Clinical features in older children include development delay and seizures, while young adults may present headaches. Clinical features and prognosis depend on the severity of the complications.

The diagnosis is usually made on prenatal ultrasound. MRI allows the assessment of the relationship between the different pathological vessels as well as the presence of complications such as hydrocephalus and brain damage. Angiography is the gold standard for full characterization and treatment of the vascular malformation, including different vascular approaches and embolic agents.
